# Accumulating evidence in ecology: Once is not enough

**DOI:** 10.1002/ece3.5836

**Published:** 2019-11-21

**Authors:** James D. Nichols, William L. Kendall, Gregory Scott Boomer

**Affiliations:** ^1^ Patuxent Wildlife Research Center U.S. Geological Survey Laurel MD USA; ^2^ Colorado Cooperative Fish and Wildlife Research Unit U.S. Geological Survey Fort Collins CO USA; ^3^ U.S. Fish and Wildlife Service Laurel MD USA

**Keywords:** Bayes theorem, ecology, evidence, information state, knowledge, replication, reproducibility, science

## Abstract

Many published studies in ecological science are viewed as stand‐alone investigations that purport to provide new insights into how ecological systems behave based on single analyses. But it is rare for results of single studies to provide definitive results, as evidenced in current discussions of the “reproducibility crisis” in science. The key step in science is the comparison of hypothesis‐based predictions with observations, where the predictions are typically generated by hypothesis‐specific models. Repeating this step allows us to gain confidence in the predictive ability of a model, and its corresponding hypothesis, and thus to accumulate evidence and eventually knowledge. This accumulation may occur via an ad hoc approach, via meta‐analyses, or via a more systematic approach based on the anticipated evolution of an information state. We argue the merits of this latter approach, provide an example, and discuss implications for designing sequences of studies focused on a particular question. We conclude by discussing current data collection programs that are preadapted to use this approach and argue that expanded use would increase the rate of learning in ecology, as well as our confidence in what is learned.

## Graphical abstract

Most scientific research papers in ecology are considered as stand‐alone constructions, whereas few ecological questions can be definitively answered with single studies. We advocate repeat tests of hypothesis‐based predictions against observations as a means of learning. Specifically, we recommend accumulating evidence using an evolving information state approach based on the predictive abilities of hypotheses.

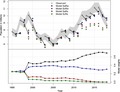

## 1 INTRODUCTION

Science has long been viewed as a progressive endeavor in which knowledge accumulates through time via the collective efforts of multiple investigators. For example, in 1637 Descartes wrote: “I hoped that each one would publish whatever he had learned, so that later investigations could begin where the earlier left off” (Descartes, 1960). A superficial look at the recent explosion of scientific journals and published studies therein might suggest that knowledge is now accumulating quite rapidly. However, clear thinkers have periodically reminded us that large numbers of studies and associated findings do not necessarily reflect successful accumulation of knowledge. Poincare (1905) wrote: “Science is built up of facts, as a house is with stones. But a collection of facts is no more a science than a heap of stones is a house.” Extending this structural metaphor in his essay “Chaos in the Brickyard,” Forscher (1963) wrote of the “edifice” of accumulated knowledge, built with the “bricks” of individual study results. Forscher (1963) warned that the bricks were becoming ends unto themselves and ended his essay with the comment: “And, saddest of all, sometimes no effort was made even to maintain the distinction between a pile of bricks and a true edifice.” We contend that most ecological investigations are viewed as stand‐alone studies, with inadequate attention devoted to accumulation of evidence and subsequent knowledge. This worry has consequences for the related applied disciplines of conservation and wildlife management as well. In this essay, we describe our view of the status quo in ecological science, document problems with the current approach, describe approaches to accumulating evidence, and then make recommendations for increased emphasis on accumulation of evidence.

Throughout this paper, we discuss the accumulation of “evidence” and “knowledge.” We view these two terms as closely related, but not interchangeable. Our operational definition of “evidence,” for the purpose of this particular paper, is the degree of consistency of observations with predictions based on a priori hypotheses. These predictions are usually generated by mathematical models developed to represent the parent hypotheses. Accumulated evidence refers to multiple tests or analyses directed at the same hypothesis in which consistency between observations and predictions is observed. We operationally define “knowledge” as confidence in the predictive ability of a hypothesis developed through accumulated evidence. We do not define knowledge relative to unknowable truth. Instead, it reflects provisional human understanding of how some natural process or system works, as assessed via predictive ability. That understanding is conditional on the set of hypotheses developed for that process or system.

## 2 STATUS QUO: ONE‐AND‐DONE IS WHAT WE DO

The majority of published studies in ecological journals appear to be viewed by their authors as stand‐alone investigations. This is understandable for exploratory analyses, the prevalence of which has been criticized through the years (e.g., Platt, 1964; Romesburg, 1981; Yoccoz, Nichols, & Boulinier, 2001). The primary role of such studies in science is hypothesis generation, so at best they represent a starting point for any accumulation of evidence.

In this paper, we focus on studies that seek to test predictions of a priori hypotheses and contend that most of these are viewed by their authors as stand‐alone endeavors (Murad & Montori, 2013). *Introduction* sections of papers often cite previous papers that deal with the focal subject, but frequently for the purpose of (a) emphasizing the inadequacies in previous work as motivation for the current effort, or (b) noting differences that distinguish the current work from previous efforts. Similarly, *Discussion* sections frequently cite‐related work, sometimes noting results that did and did not agree with those of the current effort. However, *Discussion* sections seldom include rigorous assessments of the degree to which results have contributed to an overall body of evidence or knowledge for the studied subject.

This emphasis on stand‐alone studies extends to the literature on statistical inference and related design issues. In criticizing “the cult of the isolated study,” Nelder (1986) wrote “Most statistical books and papers place enormous emphasis on the analysis of the unique experiment or study. Much statistical expertise is deployed to make inferences from a single isolated data set, treated as if it were essentially unique.” Inference methods for animal population dynamics focus on estimating key parameters based on single data sets (e.g., Kery & Royle, 2015, in press; Seber, 1982; Williams, Nichols, & Conroy, 2002). Most of the literature on model selection is similarly focused on candidate models fit to single data sets (e.g., Burnham & Anderson, 2002; Hooten & Hobbs, 2015; Link & Barker, 2006). Texts on statistical design reflect the single study emphasis, typically focusing on a design criterion (e.g., maximize test power) for single experiments or sets of observations (see Nelder, 1986). In contrast, we have seen relatively few formal efforts to draw inferences from evidence accumulated across multiple studies, and very few to design sequences of studies with a focus on accumulated evidence (but see Chaloner & Verdinelli, 1995; Dietze et al., 2019; Hooten, Johnson, & Brost, 2019). “This emphasis on the isolated study, with the corresponding lack of emphasis of problems of combining information from many experiments, is, I believe, an unsatisfactory feature of much statistical writing” (Nelder, 1986). We agree.

## 3 MOTIVATION: ONCE IS NOT ENOUGH

We believe that there are few important questions in ecology or conservation that can be definitively answered with a single study, for example via one of Platt's (1964) “crucial experiments.” Although single experiments in ecology sometimes yield definitive results (e.g., Paine, 1976), these are uncommon. The common inability to conduct true manipulative experiments in ecology (especially for vertebrate field studies and the large spatial scales that they require) distinguishes it from other disciplines such as biomedical research, for which well‐designed clinical trials can be viewed as gold standards (Begley & Ioannidis, 2015). Hypotheses in ecology tend to be complex relative to those in some other disciplines, such that it is unusual to hypothesize a single posited cause as both necessary and sufficient for a system response. Instead, most hypotheses in ecology are multifactorial (e.g., Hilborn & Stearns, 1982; Lidicker, 1991), leading to a focus on relative contributions of factors to focal responses. In addition, ecologists frequently note “context dependence” of results, in which key factors elicit system responses in some situations but not others. This idea of context dependence is related to the century‐old discussion of hidden or lurking covariates (e.g., Fisher, 1958; Yule, 1903) and includes the important possibility of treatment by covariate interactions. Context dependence is an omnipresent possibility in observational studies, which dominate many areas of ecological research.

As noted above, most of the recommended approaches to model selection focus on candidate models fit to a single data set, with selection statistics based on model fit to the data and model parsimony (e.g., Burnham & Anderson, 2002; Hooten & Hobbs, 2015; Link & Barker, 2006). Recent discussions of model selection have emphasized the utility of “out‐of‐sample” prediction (Hooten & Hobbs, 2015), and our recommendation is to extend this thinking beyond the data in hand, to data not yet collected. This emphasis is consistent with traditional views of science (e.g., Chamberlin, 1897; Platt, 1964; Popper, 1959) in which hypotheses are subjected to repeated tests using new data independent of those used to create the associated model(s).

A compelling motivation for focusing on accumulation of evidence and knowledge based on multiple studies is provided by investigations of scientific reproducibility carried out over the last 15 years in multiple disciplines (e.g., Begley & Ioannidis, 2015; Ioannidis, 2005; Open Science Collaboration, 2015). In the case of biomedical research, 75%–90% of preclinical research results published in quality outlets could not be reproduced in subsequent studies (Begley & Ioannidis, 2015). When observational studies were considered, results were even worse, with 0 of 52 predictions of such studies confirmed in randomized clinical trials (Begley & Ioannidis, 2015; Young & Karr, 2011). Such results prompted Young and Karr (2011) to assert: “Any claim coming from an observational study is most likely to be wrong.” These findings should be of great concern, as observational studies, as well as related quasi‐experimental and constrained‐design studies, are so common in ecology. In the field of psychology, replication of 100 studies found that replication effects were half the size of original study effects, and only 39% of these effects were judged to have replicated original results (Open Science Collaboration, 2015). There is growing recognition that reproducibility is likely to be an important problem in ecology as well (Ellison, 2010; Fidler et al., 2017; Ives, 2018; Parker et al., 2016; Schnitzer & Carson, 2016). The lead entry in a list of proposals developed by Begley and Ioannidis (2015) to deal with the reproducibility crisis and improve quality of scientific research was for editors to solicit replication bids, rewarding investigators willing to undertake serious efforts at replicating published results (Wagenmakers & Forstmann, 2014).

Our emphasis here is on the science of ecology, but we note that these various problems with relying on results from stand‐alone studies also extend to the applied disciplines based on ecological science. Specifically, management actions and policy development may be based on nonreproducible results of single studies, resulting in wasted time and management effort expended on conservation problems (e.g., Walsh, Wilson, Benshemesh, & Possingham, 2012). We suspect that this kind of problem is widespread in conservation and wildlife management.

## 4 APPROACHES TO ACCUMULATING EVIDENCE

Given these problems associated with a discipline dominated by one‐and‐done studies, how can we begin to pay more attention to the accumulation of evidence in ecology and conservation? Methodological approaches that have been used in ecology can be categorized as ad hoc, meta‐analyses, and evolving information state.

### 4.1 Ad hoc

Prior to the 1990s, ecological science was dominated by hypothesis‐testing approaches, and these are not uncommon today. Studies usually entail a focal hypothesis tested against either a null or an omnibus alternative. Under this approach, ecologists develop increased confidence in hypotheses that withstand repeated efforts at falsification (e.g., Popper, 1959, 1963). Popper (1959, 1972) introduced the biological analogy of natural selection of hypotheses in which some hypotheses survive falsification efforts and many do not. Such an approach leads to a set of hypotheses that survive and become our provisional ecological knowledge, and many that do not and are discarded.

This approach to accumulating knowledge has been prevalent in ecology and has led to most of the theories and even laws that appear in ecological texts. The approach requires that different investigators subject some of the same basic hypotheses to tests in order to provide the repeated testing that can engender confidence. This reliance on independent decisions by ecologists about what to study does not lend itself to designing programs to accumulate evidence, but the approach has seemed to “work” for ecology in a general way.

We note the special cases of long‐term ecological studies in which single investigators or groups study a particular system over a long period of time (e.g., Cooke, Rockwell, & Lank, 1995; Rotella, Link, Chambert, Stauffer, & Garrott, 2012; Spendelow et al., 2016). Such long‐term efforts nearly always investigate multiple hypotheses, but they also revisit past study results by comparing new observations with model‐based predictions. Because such investigations are often led by the same investigators or teams, there is an increased tendency to incorporate repeated tests into study design, providing an opportunity for faster, directed accumulation of evidence and consequent learning.

### 4.2 Meta‐analyses

Meta‐analyses represent attempts to assess accumulated knowledge at specific points in time and were adapted from other disciplines by ecologists in the early 1990s. The term “meta‐analysis” has been used in multiple ways, with one definition: “the statistical analysis of a large collection of analysis results from individual studies for the purpose of integrating the findings” (Glass, 1976:3). Meta‐analyses of this type are conducted using results from multiple published papers focusing on a specific question (Gurevitz, Curtis, & Jones, 2001; Gurevitz, Koricheva, Nakagawa, & Stewart, 2018; Korichava, Gurevitz, & Mengersen, 2013). Summary statistics from selected papers are subjected to secondary analysis intended to provide an overall inference. These summary statistics may be test statistics, their associated probability levels, or estimates of effect sizes and their associated variances. Such meta‐analyses are useful in providing assessments of accumulated evidence at specific points in time (Gurevitz et al., 2018), and they have seen use in both ecology (e.g., Korichava et al., 2013) and conservation (e.g., Walsh et al., 2012).

Such meta‐analyses rely on available published studies and hence can suffer from the various forms of selection and interpretation bias that characterize published research (e.g., Begley & Ioannidis, 2015; Fidler et al., 2017; Gurevitz et al., 2018; Palmer, 1999; Whittaker, 2010). Integration of results of multiple studies with different study scales, designs, sources of bias, and degrees of relevance to the focal hypothesis can be a complex task. The biomedical community has devoted substantial attention to this issue, developing several promising approaches for dealing with different sources of study bias, for example (Turner, Spiegelhalter, Smith, & Thompson, 2009). Nonetheless, the following claim by Ioannidis (2016) is sobering: “Few systematic reviews and meta‐analyses are both non‐misleading and useful.” Recent calls for transparency in reporting ecological research have been directed largely at increasing the utility of published investigations for research summaries and meta‐analyses (Ellison, 2010; Fidler et al., 2017; Parker et al., 2016; Schnitzer & Carson, 2016). Importantly from the perspective of this essay, the opportunistic nature of most meta‐analyses precludes design of component studies and does not provide natural opportunities for designing sequences of studies. The biomedical research community has begun to use the concept of value of information (Raiffa & Schlaifer, 1961) as a basis for design of future studies (Ades, Lu, & Claxton, 2004; Jackson, Presanisa, Contib, & Angelisa, 2019).

Another kind of meta‐analysis entails periodic modeling of long‐term data sets from multiple study locations, as practiced for spotted owls (*Strix occidentalis*) in the western United States (e.g., Anthony et al., 2006; Blakesley et al., 2010; Dugger et al., 2016; Forsman et al., 2011; Franklin et al., 2004). Not only are analyses based on multiple study sites, but they are also repeated periodically, with each successive analysis based on a longer time series of data. The spotted owl meta‐analyses were originally focused on estimation of trend statistics, rather than on evaluating mechanistic hypotheses. However, the planned periodic nature of these meta‐analyses should permit design focus on accumulation of evidence associated with selected hypotheses.

### 4.3 Evolving information state

A more formal approach to accumulating evidence is based on a multiple‐hypothesis approach to the conduct of science (e.g., Burnham & Anderson, 2002; Chamberlin, 1897). As with the Popperian single‐hypothesis approach to science, the key step in multiple‐hypothesis science is the comparison of observations against model‐based predictions for the different hypotheses being considered. However, instead of rejecting or provisionally accepting a single hypothesis (e.g., relative to a null), inferential results can be presented as model “weights” reflecting the relative (to other models in the considered set) degree of correspondence between observations and predictions, and sometimes model parsimony as well. Information–theoretic methods of model selection and multimodel inference for single analyses were introduced to ecologists in the early 1990s (e.g., Burnham & Anderson, 1992; Lebreton, Burnham, Clobert, & Anderson, 1992) and have become widely adopted in ecology, conservation, and various other disciplines (Burnham & Anderson, 2002 has been cited >45,000 times). Bayesian approaches to model selection have begun to see substantial use in ecology as well (Barker & Link, 2013; Brooks, Catchpole, Morgan, & Harris, 2002; Hooten & Hobbs, 2015; Link & Barker, 2006, 2009). However, these general applications of a multiple‐hypothesis approach in ecology and elsewhere have been largely focused on single studies and not on the accumulation of evidence across studies.

The information state approach to accumulating evidence is based on the relative performance of multiple models (hence multiple hypotheses) in predicting observations obtained during a sequence of comparisons or studies conducted in either the same or multiple locations. This approach has been advocated in fisheries and wildlife sciences since the mid‐1970s (Hilborn & Walters, 1992; Johnson et al., 1993; Walters, 1986; Walters & Hilborn, 1976, 1978; Williams, 1996; Williams et al., 2002; Williams, Szaro, & Shapiro, 2007), and more recently in ecology (Dietze et al., 2019; Hilborn & Mangel, 1997), but has seen very little use. Model weights comprising the information state do not reflect both model parsimony and fit to a single data set, as in single study model selection. Instead, the information state at any time *t* carries the results of a sequence of observations or studies conducted prior to *t*, with model‐specific weights reflecting the degree to which model‐based predictions have been consistent with past observations of the sequence. As observations from a new (*t* + 1) comparison or study become available, their consistency with model‐based predictions is assessed and then combined with prior model weights via Bayes' theorem to update model weights with the new information (Box 1). Updated model weights are larger than previous weights for models that predicted well and smaller for models that predicted poorly. Model weights are scaled to sum to 1 (or to integrate to 1 for model sets expressed as a continuum) for all of the models in the set. If one of the models in the set approximates the underlying process well and is a good predictor, then its weight should approach 1 over time, whereas the weights of relatively poor predictor models should eventually approach 0.

Box 1 **Model weight updating with Bayes' Theorem**

Define the “information state” as a vector of model weights, *π_t_* (model *i*) for model *i* at time *t*, that reflect the relative predictive abilities of models in the model set. In the case of *M* models in the set:

(1) $$\sum_{i=1}^{M}\pi_{t}\left(\text{model}\;i\right)=1$$

We have more confidence in the models with higher weights and view them as more likely to represent reasonable abstractions of the modeled natural processes. Initial weights prior to the first set of observations can be based on historic information, intuition, or simply set equal (1/*M*) for each model.

Subsequent model weights typically change with each new set of observations, evolving according to:

(2) $$\left(\pi_{t+1}\left(\text{model}\;i\right)|{\text{data}}_{t+1}\right)=\frac{\pi_{t}\left(\text{model}\;i\right)\mathrm{Pr}\left({\text{data}}_{t+1}|\text{model}\;i\right)}{\sum_{j=1}^{M}\pi_{t}\left(\text{model}\;j\right)\mathrm{Pr}\left({\text{data}}_{t+1}|\text{model}\;j\right)}$$

where Pr (data*_t_*_+1_|model *i*) is the probability that the new observations at time *t* + 1 would have arisen, given that model *i* was a good representation of the actual process that generated them. The updating of model weights is based on the relative confidence in the model that has accumulated through time *t*, *π_t_* (model *i*), and the consistency of the new set of observations with that model, Pr (*data_t_*_+1_|model *i*). If the model set includes a good approximating model that predicts reasonably well, then the weight for that model should evolve to approach 1, whereas the weights of models that predict more poorly should eventually approach 0.

If the model set includes no models that are reasonable approximations to underlying processes, then we do not expect model weights to evolve as described above. Instead, nonmonotonic fluctuations in model weights may be indicative of a need for additional, better models. Often, the directions of differences between model‐based predictions and observations provide clues to the sorts of new model components that may be needed. Temporal changes in predictive abilities of models (e.g., becoming less predictive over time) may indicate the need for additional model components that deal with global change (e.g., Nichols et al., 2011; Zhao, Silverman, Fleming, & Boomer, 2016). Periodic assessments of the evolution of the information state are useful and may lead to deletion of some models and insertions of new ones. Although decisions are required about adjusting and setting model weights immediately after such changes to the model set, the described process readily admits such changes.

**Figure 1:**
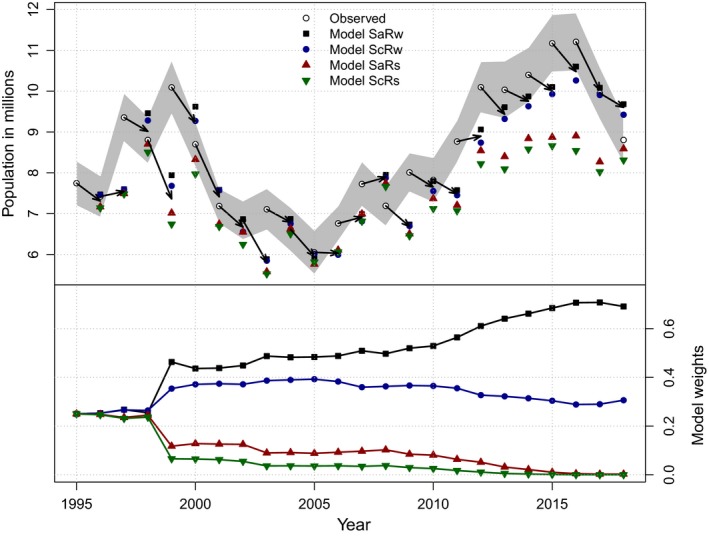
Upper panel: population estimates of mid‐continent mallards (in millions) compared to predictions of each member of the model set (SaRw = additive mortality and weakly density‐dependent reproduction, ScRw = compensatory mortality and weakly density‐dependent reproduction, SaRs = additive mortality and strongly density‐dependent reproduction, ScRs = compensatory mortality and strongly density‐dependent reproduction). The gray shading represents 95% confidence intervals for observed population estimates. The arrow represents a weighted mean annual prediction based on the entire model set. Lower panel: annual changes in model weights for each member of the mid‐continent mallard model set; weights were assumed to be equal in 1995

Figure 1 shows the evolution of model weights for an actual example from 23 years of study in the applied ecological sciences (Box 2). In this example, initial model weights were set equal for four competing hypotheses and annually updated based on comparisons of model‐based predictions with population size estimated from an extensive monitoring program. 2018 model weights are relatively small for two of the hypotheses, but some uncertainty remains for the two remaining hypotheses. The evolution depicted in Figure 1 is based on a system manipulated to achieve management objectives, rather than scientific objectives. More rapid evolution is expected when the focal system is manipulated for the purpose of facilitating model discrimination. Note also that the four hypotheses were based on effects of hunting and density on the vital rates survival and reproduction, respectively, whereas monitoring data used in Equation 1 were estimates of the state variable, population size. Changes in population size represent the integrated effects of factors such as hunting and density on survival and reproduction, making discrimination among competing models more difficult than if the vital rates themselves had been estimated and compared with predictions. Despite these two handicaps, model discrimination was possible over the 23‐year sequence of observations (Figure 1).

Box 2 **Mid‐continent mallard (*Anas platyrhynchos*) harvest management in North America**

The U.S. Fish and Wildlife Service (FWS) sets annual hunting regulations for harvested migratory birds in the United States. Regulations are sometimes contentious, with some stakeholders favoring restrictive regulations and others favoring very liberal regulations. In the 1960s and early 1970s, these diverse views led to arguments about appropriate hunting regulations for mallard ducks, fueled by substantial uncertainty about population‐level effects of hunting (Nichols, 2000a). As a result, seminal research was conducted to develop analytical methods to determine the impact of harvest mortality on waterfowl populations (e.g., Anderson & Burnham, 1976; Burnham & Anderson, 1984). Comprehensive efforts to apply these techniques to existing large‐scale data sets led to mixed results with some evidence supporting the compensatory harvest mortality hypothesis (Anderson & Burnham, 1976; Burnham & Anderson, 1984). Over time, these analyses were repeated with updated datasets, and newly developed methods were applied, but the results were still equivocal (Sedinger & Rexstad, 1994; Smith & Reynolds, 1992). In response to this continued uncertainty, researchers performed meta‐analyses to systematically review these studies, in an effort to synthesize the evidence describing the relationship between harvest mortality and survival (Nichols, Conroy, Anderson, & Burnham, 1984; Nichols & Johnson, 1996; Pöysä, Elmberg, Gunnarsson, Nummi, & Sjöberg, 2004). Given the inferential limitations to these approaches and the ambiguous results, managers had to develop harvest regulatory decisions in the face of substantial uncertainty.

The inability of ad hoc and meta‐analytical approaches to reduce key uncertainties critical to regulatory decisions led the harvest management community to apply the principles of adaptive management (Walters, 1986) to harvest decisions. Under the leadership of a FWS scientist (F. A. Johnson), a program for adaptive harvest management (AHM) was formally adopted in 1995 (Johnson et al., 1993, 1997; Nichols, Johnson, & Williams, 1995; Williams, Johnson, & Wilkins, 1996). This framework provides a means of making decisions in the face of uncertainty, while learning about population responses to harvest decisions via the use of the evolving information state. AHM has been operational for >20 years and is viewed by many observers as an important success story (Johnson, 2011; Johnson, Boomer, Williams, Nichols, & Case, 2015; Nichols, Johnson, Williams, & Boomer, 2015; Nichols, Runge, Johnson, & Williams, 2007; U.S. Fish & Wildlife Service, 2018).

The AHM approach to resolving uncertainty is based on the evolving information state as described in the text and Box 1. During the summer of each year, an optimal regulatory decision for mallard harvest regulations is identified with dynamic optimization (e.g., Bellman, 1957; Puterman, 1994; Williams, 1996) based on weighted projections of system responses from multiple models. Given an observation of the current system state (mallard spring breeding population size and the amount of breeding habitat, Smith, 1995), the appropriate hunting regulation is selected. Each model is then used to predict the population size for the following spring. Model weights are then updated with Equation 2, comparing model‐based predictions with observed abundance. Weights increase for models that perform well and decrease for those that predict poorly. These weights are then incorporated into the derivation of the next optimal regulatory decision, ensuring that the updated information state informs the next decision.

Four models of system response are used in the AHM program for mid‐continent mallards based on contrasting hypotheses about survival and reproduction. Annual survival rate is modeled under an additive (Sa; additive instantaneous competing risks) or compensatory (Sc) mortality hypothesis (Anderson & Burnham, 1976; Cooch, Guillemain, Boomer, Lebreton, & Nichols, 2014; Johnson et al., 1997). Annual reproductive rate is modeled as strongly (Rs) or weakly (Rw) density‐dependent (Johnson et al., 1997; U.S. Fish & Wildlife Service, 2018). Combined, these hypotheses result in four models: SaRs (additive mortality, strongly density‐dependent reproduction), SaRc (additive mortality, weakly density‐dependent reproduction), ScRs (compensatory mortality, strongly density‐dependent reproduction), and ScRw (compensatory mortality, weakly density‐dependent reproduction). In 1995, initial model weights were assigned to be equal at 0.25 for each of these models. After 23 years of experience with AHM, model weights have evolved based on model‐specific predictions, with hypotheses reflecting strongly density‐dependent reproduction (SaRs, ScRs) showing decreases in model weight reflecting low relative predictive ability (Figure 1). Of the two hypotheses including weakly density‐dependent reproduction, the one with additive mortality (SaRw) has the largest model weight, but ScRw has a non‐negligible model weight as well. Figure 1 reflects learning about mallard responses to hunting and is thus useful to management. Evolving model weights reflect changes in which models have most influence on each year's hunting regulations, with SaRw and to a lesser extent ScRw dominating the optimization at present.

The models of Figure 1 represent distinct hypotheses about how variation in hunting mortality affects population dynamics. The component hypothesis of strongly density‐dependent reproductive rate was represented by a single model structure (Box 2) in the 4‐model set. However, different structural forms of this basic hypothesis could have been incorporated into the model set also, as different forms may indeed lead to different management decisions in some cases (Runge & Johnson, 2002). Thus, the approach of the evolving information state can be used to discriminate among models that represent very different hypotheses or simply different forms of a single hypothesis. Of course the more similar the predictions of different hypotheses, the more difficult it will be to discriminate among them, regardless of the approach used.

The use of an approach to accumulating evidence based on the evolving information state provides a good response to the motivating arguments discussed in the previous section. For example, model selection can be based on model performance in repeated predictions, and reproducibility is assessed periodically with each confrontation of observations and model‐based predictions (e.g., Ioannidis, 2005). The implementation of an evolving information state in our mallard example occurred in direct response to the failure of ad hoc approaches and crude meta‐analyses to resolve arguments about competing hypotheses important to management (Box 2). Gurevitz et al. (2018) suggested that meta‐analyses have caused authors to view each individual study “as a contribution toward the accumulation of evidence rather than revealing the conclusive answer to a scientific problem.” Although this view may be held by some ecologists, our reading of the current ecological literature leads us to doubt the generality of this perspective. The role of each comparison of data against model‐based predictions is acknowledged explicitly in the evolving information state approach.

A major advantage of the information state approach over ad hoc and meta‐analytic approaches to accumulating evidence is the potential to optimize study design. We operationally define a “program of inquiry” as a sequence of studies designed to discriminate among the competing models of a specified model set. A typical objective for a single study with two hypotheses would be to maximize test power. For the case of multiple (>2) competing hypotheses, a reasonable objective would be to minimize a diversity index based on model weights (see Box 3), where minimization would reflect a weight approaching 1 for a single model and weights approaching 0 for the remaining models.

Box 3 **Shannon entropy as an optimization criterion for programs of inquiry**

Test power, a common optimization criterion for the planning of hypothesis tests based on two hypotheses, is not so appropriate for designing studies with multiple (>2) hypotheses. Instead, some criterion based on the model weights, *π_t_* (model *i*), would be preferable (see Box 1). One approach is to consider the diversity of the model weights, where high diversity indicates relatively even model weights and low diversity indicates greater confidence in one or more models and less confidence in the remainder.

**Figure 2:**
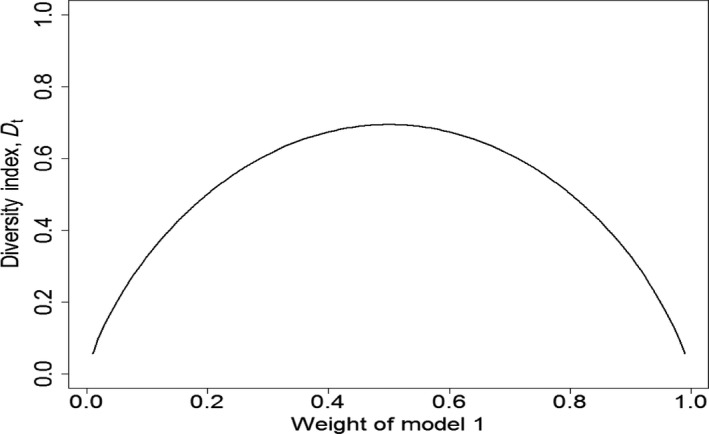
Shannon entropy (Equation 3) computed for varying model weights in the case of two models

A commonly used diversity index is Shannon entropy (Shannon, 1948) computed using natural logs (ln):

(3) $$D_{t}=-\sum_{i=1}^{M}\pi_{t}\left(\text{model}\;i\right)\ln\left[\pi_{t}\left(\text{model}\;i\right)\right]$$

where *D_t_* is Shannon diversity at time *t*, *M* denotes the number of models in the set, and *π_t_* (model *i*) is the weight for model *i* at time *t*. As noted, *D_t_* would not necessarily be used for the case of only two models, but we plot *D_t_* as a function of model weight for one of two models simply for ease of presentation and understanding (Figure 2). In the case of two models (Figure 2), *D_t_* attains its highest value for *π_t_* (model 1) = *π_t_* (model 2) = 0.5 and then approaches 0 for *π_t_* (model *i*) approaching 0 or 1. Thus, low diversity is indicative of selection of an appropriate model, whereas high diversity is indicative of substantial uncertainty, with little discrimination among models.

Study design for such a sequence of studies can be viewed as a dynamic Markov decision problem (Puterman, 1994; Williams et al., 2002) where the decision can take the form of a study treatment, a management action, a set of observations to collect, etc. This approach requires projecting the information state forward through time, accomplished in this case through use of Equation 2 (Box 1). Framing study design in this manner allows the decision at any decision point, *t*, to be a function of information state at that time. This is important, as certain aspects of optimal study design are expected to vary depending on model weights (Box 4). This dependence of design on information state should seem intuitively reasonable, as different distributions of uncertainty across models should lead to differing approaches to discrimination. Formal study objectives could be to minimize terminal (final time step of study) diversity, minimize time‐averaged diversity, or perhaps minimize time required to achieve a threshold diversity value (e.g., *D_t_* < 0.05). The focus of the dynamic decision problem would be to select appropriate actions. For manipulative studies, actions would be such factors as whether, how, and where (in the case of multiple study locations) to manipulate the system (Box 4). For both manipulative and observational studies, actions would include selection of parameter(s) to estimate, selection of estimation method, selection of sample size, etc. Such treatment of study design for sequential programs of inquiry has the potential to increase the rate of learning and should receive greater consideration, in our opinion.

Box 4 **Example of optimal study treatment as a function of current information state**

If accumulation of evidence via the evolution of model weights is pursued, and a series of studies is designed to maximize that rate of accumulation, then each study will be designed to maximize discrimination among models (see Boxes 1 and 3). We consider the design of an experiment to evaluate the distributional dynamics of a species, based on its local extinction and colonization processes. Competing hypotheses about the system dynamics are expressed through four models, with the following values for baseline extinction (*e*) and colonization (*c*) probabilities: Model 1: *e* = 0.3, *c* = 0.3; Model 2: *e* = 0.3, *c* = 0.4; Model 3: *e* = 0.1, *c* = 0.3; Model 4: *e* = 0.1, *c* = 0.4. There are 50 sites for which presence of the species is possible. There are 40 sites currently occupied. Two treatments are considered: (a) do nothing or (b) eradicate the species from 20 of the occupied sites.

For each candidate treatment, and conditional on the current state of the system (*O_t_* occupied sites and *U_t_* = 50 − *O_t_* unoccupied sites), the diversity index in Box 3 is computed using the expected post‐treatment weight ($E\left(\pi_{t+1}\left(\text{model}\;i\right)\right)$) for each of the four models, summed over all possible resultant extinctions (*E_t_*_+1_) and colonizations (*C_t_*_+1_). Given each possible resulting combination of *E_t_*_+1_ and *C_t_*_+1_, a new predicted weight for each model *j* (i.e., new information state) can be computed, using Equation 2. In this case Pr (data*_t_*_+1_|model *j*) = Pr (*E_t_*_+1_, *C_t_*_+1_|model *j*) = Pr (*E_t_*_+1_|model *j*) × Pr (*C_t_*_+1_|model *j*), assuming the extinction and colonization processes are independent. The probabilities of the resulting *E_t_*_+1_ and *C_t_*_+1_, respectively, for model *j* are based on independent binomial distributions with *O_t_* and *U_t_* trials, respectively, and success probabilities *e_j_* and *c_j_*, respectively. Each of these new system state‐dependent information states is then weighted by the average probability of reaching that new state ($\sum_{j=1}^{4}\pi_{t}\left(\text{model}\;j\right)\mathrm{Pr}\left(E_{t+1},C_{t+1}|\text{model}\;j\right)$). The final expected posterior weight for model *i* is then derived by summing across all possible resulting extinctions and colonizations:

$$\begin{array}{c} E\left(\pi_{t+1}\left(\text{model}\;i\right)\right)\\ =\sum_{E=0}^{\text{occupied}}\sum_{C=0}^{\text{unoccupied}}\left[\sum_{j=1}^{4}\pi_{t}\left(\text{model}\;j\right)\mathrm{Pr}\left(E_{t+1},C_{t+1}|\text{model}\;j\right)\right]\pi_{t+1}\left(\text{model}\;i|\left\{E_{t+1},C_{t+1}\right\}\right) \end{array}$$

**Figure 3:**
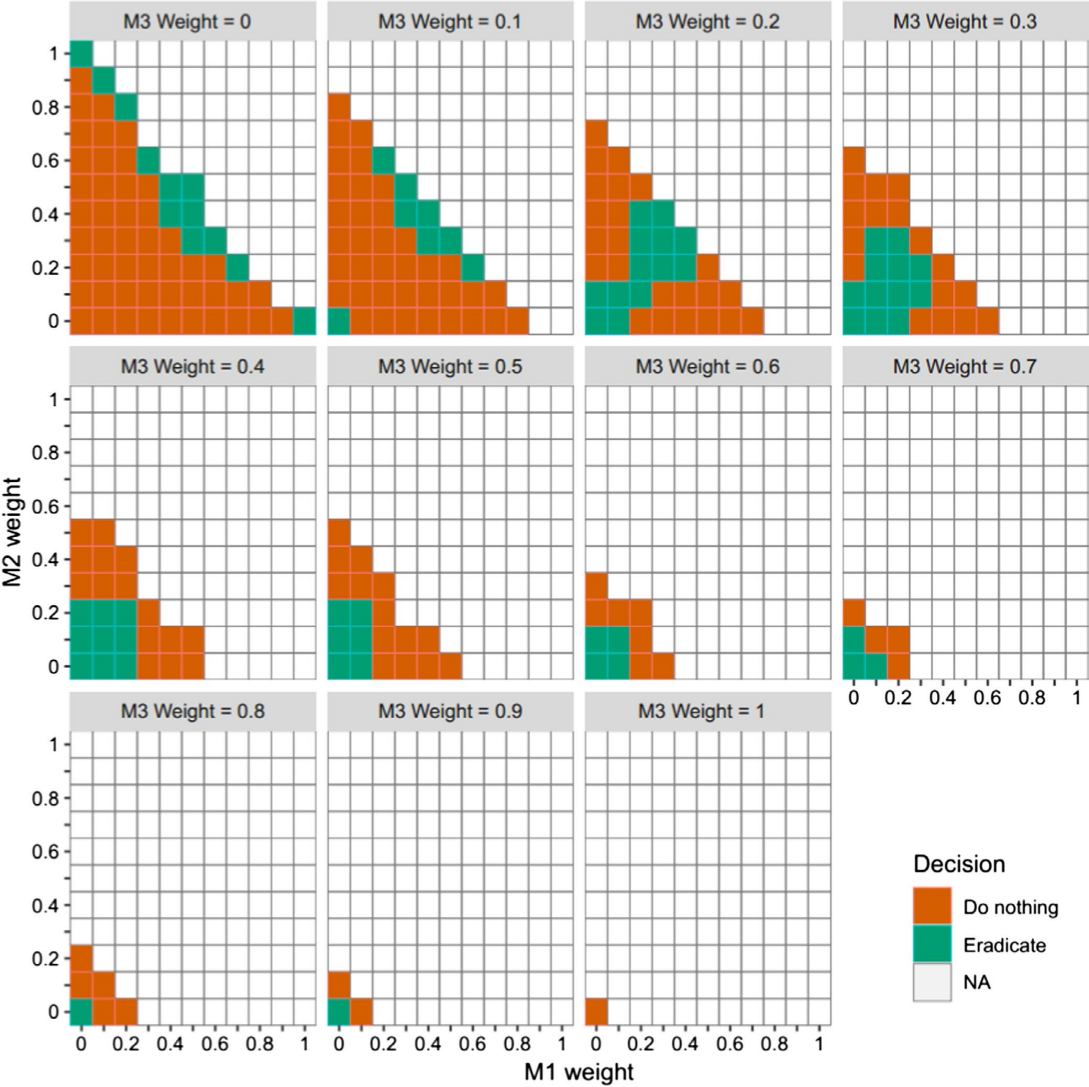
The results of an optimal design approach to treatment selection for one time step of an experiment on distributional dynamics. Hypotheses about system dynamics are expressed using four models with differing values for extinction (*e*) and colonization (*c*) probabilities: Model 1: *e* = 0.3, *c* = 0.3; Model 2: *e* = 0.3, *c* = 0.4; Model 3: *e* = 0.1, *c* = 0.3; Model 4: *e* = 0.1, *c* = 0.4. There are 50 experimental sites, 40 of which are currently occupied. Two treatments are considered: (a) do nothing; or (b) eradicate the species from 20 of the occupied sites. Figure depicts optimal treatment for all possible initial weights for each model, in increments of 0.1

The results of this optimal design approach for one time step, with initial system state of 40 out of 50 sites occupied, are shown in Figure 3, for all possible initial weights for each model, in increments of 0.1. The optimal treatment depends on the current information state. Treatment 2, the eradication of 20 sites, tends to be selected in the upper right or lower left of each plot. These are scenarios where there is less uncertainty about the extinction process, and therefore, it is advantageous to create more unoccupied sites, in order to examine the colonization process.

The first ecological recommendations for use of the evolving information state approach to accumulating evidence came from applied ecology in conjunction with the concept of adaptive management (e.g., Hilborn & Walters, 1992; Johnson et al., 1993; Walters, 1986; Walters & Hilborn, 1976, 1978; Williams, 1996; Williams et al., 2002; Williams et al., 2007). In addition to the management program described in Box 2, this adaptive management approach with evolving information state is being used in a small number of other management programs (Martin et al., 2011; McGowan et al., 2015; U.S. Fish & Wildlife Service, 2018). Active adaptive management incorporates projected changes in the information state into periodic decision‐making (Williams, 2011) and is thus closely related to the active study design approach to learning described above. Passive adaptive management includes changes to the information state as a by‐product of management and thus still includes the updating of model weights and consequent learning, but not the design opportunities (Williams, 2011). Recently, Dietze et al. (2019) advocated increased use of iterative near‐term forecasting and adaptive management as means of developing predictive ability useful to decision‐makers. When uncertainty about system responses to management actions leads to use of multiple models and ensemble predictions, the information state provides a natural way to weight models for such predictions.

For clarity, we distinguish between the described sequential approach using an evolving information state and the single‐analysis approach of using AIC model selection for a single data set that includes the same multiple‐period data. As emphasized in the above discussion of study design, one distinction is the ability to adapt design components (e.g., which system manipulations to impose or which data to collect and parameters to estimate) as appropriate when using the multiple assessments of the evolving information state. Beyond this, the usual model selection approach with a single data set is to fit models to the entire data set. The models contain general parameters for which maximum‐likelihood estimates (MLEs) are obtained. Efforts to evaluate model fit are based on observed and expected values for selected statistics, where expected values are computed using the same data as used to compute MLEs. In the evolving information state approach, parameter values are specified initially, or at least before the new data are collected, based on either theory or prior data. Utility of each model is judged by comparing predictions against data and estimates from the next time step, which are independent of the specified parameter values of each model. This approach avoids potential circularity associated with use of the same data for both assessment and estimation of parameter values.

Although the evolving information state approach was not developed with system change in mind, a shift from one model best describing dynamics to another (as possible under some climate change scenarios) would likely be apparent in the trajectory of model weight evolution. In contrast, a single analysis of the entire data set is expected to produce MLEs and model weights that reflect average (over the entire data set) values. Finally, primary motivations for seeking a parsimonious model using approaches such as AIC are to obtain parameter estimates with smaller variances and to guard against “overfitting,” such that the focal data set is fit extremely well, but the selected model is not useful for prediction (Burnham & Anderson, 2002). By focusing on predictive abilities, the evolving information state approach addresses this latter issue directly. In sum, we do not know how frequently these two approaches to model selection would yield the same models, but these distinctions between the approaches provide the potential for different selections.

The described approach of an evolving information state is based on the classical framework of discrete models that provide different stories about how the world works. We note the existence of a closely related approach that focuses not on model weights for discrete models, but on estimation of key parameters in a single general model. For example, in the mallard example of Box 2, reproductive rate was modeled as either strongly or weakly density‐dependent. We could have developed a single model with one parameter describing the strength of density dependence and focused on its estimation. Under this approach to modeling, the accumulation of evidence becomes a problem of updating estimates of key parameters as new data become available. Methods referred to as Recursive Bayes provide a natural approach for this sort of updating (see Hooten et al., 2019), although we still recommend the scientific step of comparing predictions with observations as a means of determining whether the general model itself is providing a reasonable approximation of underlying ecological processes (see Dietze et al., 2019). Thus, other approaches to accumulating evidence may be useful, in addition to the one that we propose.

In summary, we believe that the evolving information state is a good approach to accumulating evidence that deserves far more extensive use. It provides a scientific and systematic approach to accumulation of evidence. The model weights that form the information state are objectively based on the ability of each model to repeatedly make predictions that are consistent with observations. Models that attain high weights evoke confidence, not because of loud or influential advocates, but simply because they predict well. We believe that more widespread use of this approach could lead to reductions in the sorts of posturing and arguing that sometimes characterize contentious debate. Finally, we believe that the ability to better design programs of inquiry has the potential to lead to more rapid learning.

## 5 OPPORTUNITIES FOR ACCUMULATING EVIDENCE: WE CAN DO BETTER

We believe that the reasoning presented in the section on motivation argues strongly for greater efforts devoted to sequences of studies that allow us to accumulate evidence in ecology and conservation. The reproducibility crisis alone provides strong motivation for promoting sequential studies designed to replicate results. Such sequences could include multiple studies implemented on the same system over time, as with our mallard example, or studies applied to different systems, allowing for a system effect. However, we recognize that a variety of research constraints, ranging from funding to publishing to availability of research team colleagues, can make the transition away from one‐and‐done research difficult. Perhaps as a result, we are aware of no current program of nonapplied ecological research that employs this approach of an evolving information state. Here, we suggest opportunities by focusing on research and other investigative activities that are preadapted to sequential studies and accumulation of evidence.

An increasingly common endeavor in current ecology and conservation is monitoring (Likens & Lindenmayer, 2018; Yoccoz et al., 2001). Monitoring programs are preadapted to accumulation of evidence via an evolving information state. Unfortunately, many ecological monitoring programs are not guided by hypotheses and corresponding models (Ims & Yoccoz, 2018; Nichols & Williams, 2006; Yoccoz et al., 2001). However, the substantial cost and effort associated with data collection are already present in monitoring programs, and only the intellectual tasks of hypothesis identification and model development are lacking. We believe that monitoring programs can be readily adapted to accumulate evidence via an information state approach, and that the responsibility for such adaptation lies with program organizers and administrators. The nature of the identified hypotheses will of course vary with the scale of the monitoring and the interests of program organizers and science advisers. We believe that incorporation of a scientific program of learning will add substantial value to the usual monitoring products of trend detection and assessment of system status. Indeed, these latter products have been criticized as providing inadequate justification for the substantial expenditures required by monitoring (Ims & Yoccoz, 2018; Nichols, 2000b; Nichols & Williams, 2006; Yoccoz et al., 2001). The addition of evidence accumulation for relevant hypotheses to ongoing and new monitoring programs will largely eliminate the criticism that these programs lack intellectual focus on specific questions of scientific or conservation interest. Monitoring of the new Climate‐ecological Observatory for Arctic Tundra is being developed as a component of the scientific process (Ims & Yoccoz, 2018) with Bayesian updating the planned approach to accumulating evidence. Unfortunately, such programs are still rare.

The kind of meta‐analysis based on periodic modeling of long‐term data sets from multiple study locations, as practiced for spotted owl species in the western United States (e.g., Anthony et al., 2006; Blakesley et al., 2010; Dugger et al., 2016; Forsman et al., 2011; Franklin et al., 2004), is also readily adaptable to an information state approach to learning. Originally, the spotted owl studies at multiple sites were developed largely to estimate population trends. As a primary funder of this work, the U.S. Forest Service focused these geographically separated studies on estimation of a single parameter (finite rate of population increase), illustrating the potential power of a top‐down approach to promote study integration. Analyses of recent years have extended beyond this mandate to also focus on hypotheses about drivers of spotted owl population dynamics (e.g., Dugger et al., 2016). Scientists associated with these studies have already begun the intellectual development of selecting key hypotheses and constructing associated models, such that adoption of the evolving information state approach would be a natural next step.

Scientists and research teams carrying out long‐term research programs on specific study systems (e.g., Cooke et al., 1995; Rotella et al., 2012; Spendelow et al., 2016) are similarly preadapted to accumulating evidence. In conjunction with carrying out sequential one‐and‐done studies of specific questions, researchers in such programs tend to maintain population‐level monitoring as an ongoing program component. Selection of focal hypotheses and development of associated models are the primary costs associated with adding a component for accumulating evidence. As such programs are typically question‐driven to begin with, we suspect this kind of shift should be readily endorsed and relatively easy.

The above recommendations build on existing monitoring programs, but what of the individual researcher who is not already associated with such a program? We see at least 2 possibilities here. The first is based on the idea that comparisons of model‐based predictions with observations external to ongoing monitoring can be useful in modifying model weights (Fackler & Pacifici, 2014; Williams, 2015). Thus, individual scientists interested in contributing to an existing program can do so with a simple investment in understanding the models of an ongoing program and identifying the kinds of observations that could contribute to model discrimination.

The second possibility is for a group of researchers focused on specific hypotheses to form a loose consortium around the idea of accumulating evidence. In cases where researchers investigate such hypotheses within different systems, variability could be dealt with via a system‐level random effect. For example, in the 1960s and 1970s multiple mammalogists were focusing their research on different hypotheses to explain microtine population cycles (reviewed by Krebs & Myers, 1974). Proposed mechanisms based on food quality, food quantity, behavior, genetics, and predation were among the leading hypotheses of the time, and each had its proponents. Researchers carried out their individual studies focused on their favorite hypotheses. We certainly learned from their results, but they were hardly definitive (Chitty, 1996), with questions surrounding even the better‐supported hypotheses (e.g., Graham & Lambin, 2002; Hanski, Hansson, & Henttonen, 1991; Hanski & Korpimaki, 1995). But what if these researchers had gotten together and agreed on a set of models that corresponded to the various hypotheses, such that individuals' experimental and observational results contributed not just to specific favorite hypotheses, but to the model weights of the entire set? Our belief is that contentiousness would have been reduced, key experiments and observations would have been more readily identified, and learning would have been more rapid.

Individual researchers can thus contribute to the evolving information state approach to accumulating evidence. However, there is currently little incentive to do so, beyond an individual's desire to make important contributions to evidence accumulation. For example, university department chairs and laboratory directors currently appear to value stand‐alone investigations more than the degree to which a scientist has contributed to changes in model weights within an integrated program. Major funding agencies currently appear to make funding decisions largely based on potential for publishable results that are widely cited, with little emphasis on the integration of results and resultant accumulation of evidence and knowledge. If such institutions and agencies shifted focus to such accumulation, this would likely serve as an important inducement for researchers to integrate their work into programs of inquiry guided by common models. We speculate that such top‐down approaches could be very effective, but they would require more effort and greater responsibility at the funding agency level to identify focal questions and promote integrated research designed to address them.

The above discussion has focused mainly on nonapplied ecological research, and we believe that many opportunities exist in applied programs as well. We noted that a few adaptive management programs currently utilize an evolving information state as a means of tracking accumulated evidence (Martin et al., 2011; McGowan et al., 2015; U.S. Fish & Wildlife Service, 2018), and we believe that this approach could be used for many more such programs. Many programs of management and conservation are even better adapted than typical monitoring programs to use of this approach because they already include both monitoring and underlying models. The need to develop model‐based predictions at each time step in order to make a wise decision, and the existence of system monitoring for making those decisions state‐specific, provide the key ingredients for updating the information state and accumulating evidence about system responses to management actions. We believe that adding key hypotheses and an associated information state can be accomplished without major alterations of existing management practice.

As is the case for nonapplied research, individual researchers can contribute to ongoing management programs, even with isolated studies. Research that is not part of a management program, but that is designed using two or more of the hypotheses of such a program, can lead to model updating that is separate from the systematic updating internal to the program (Fackler & Pacifici, 2014; Williams, 2015). Targeted research frequently leads to results that provide greater evidence for discrimination than more standard management program monitoring, for example, by targeting relationships affecting vital rates, as opposed to system state variables. In order to be most effective, such research should be coordinated with managers of the focal program early on.

## 6 SUMMARY

We began this essay with the observation that many published studies in ecology represent stand‐alone efforts in which study conclusions are viewed as new knowledge. We then argued that single studies in ecology seldom yield definitive results and cited evidence that the majority of studies in at least some disciplines yield results that are not reproducible. These observations and arguments led to the conclusion that ecological science could benefit from more sequences of studies that repeatedly compare observations against model‐based predictions as a way of accumulating evidence and learning. We have described an approach to accumulating evidence based on an evolving information state and have argued that this approach has many benefits and is well‐suited for ecology and conservation. We have also outlined how designing a series of studies to maximize the discrimination among models could increase the rate of evidence accumulation in ecology. We see very limited use of this approach in wildlife management and conservation and believe that expanded use could benefit many more management programs. The approach is not used in ecological science to our knowledge. However, many current monitoring programs and long‐term research programs are preadapted to use of this approach and could adopt it with relatively little effort. We view the accumulation of evidence in ecology as a major issue worthy of serious consideration. In our opinion, it is time to follow Forscher's (1963) advice, to refocus our efforts on building, and thus to return efficiency and purpose to our chaotic brickyard.

## CONFLICT OF INTEREST

None declared.

## AUTHOR CONTRIBUTION

All authors developed the key ideas and contributed substantially to manuscript revisions, JDN wrote the initial draft text, WLK wrote the initial draft of text Box 4 and carried out the associated computations, and GSB wrote the initial draft of text Box 2 and carried out the associated computations.

## Data Availability

All data are available in the Knowledge Network for Biocomplexity: https://doi.org/10.5063/F10000DT (webpage https://knb.ecoinformatics.org/view/doi:10.5063/F10000DT).
